# Evaluation of risk due to chronic low dose ionizing radiation exposure on the birth prevalence of congenital heart diseases (CHD) among the newborns from high-level natural radiation areas of Kerala coast, India

**DOI:** 10.1186/s41021-021-00231-0

**Published:** 2022-01-04

**Authors:** K. R. Sudheer, P. K. Mohammad Koya, Anu J. Prakash, Ambily M. Prakash, R. Manoj Kumar, S. Shyni, C. K. Jagadeesan, G. Jaikrishan, Birajalaxmi Das

**Affiliations:** 1 Low Level Radiation Research Laboratory, Low Level Radiation Research Section (LLRRS), Radiation Biology & Health Sciences Division (RB&HSD), Bio-Science Group (BSG), Bhabha Atomic Research Centre (BARC), Beach Road, Kollam, Kerala 691 001 India; 2 Department of Paediatrics, Victoria Hospital, Kollam, 691 001 India; 3 Department of Gynaecology, Victoria Hospital, Kollam, 691 001 India; 4 Department of Paediatrics, Taluk Head Quarters Hospital, Karunagapally, 690 544 India; 5 Department of Gynaecology, Taluk Head Quarters Hospital, Karunagapally, 690 544 India; 6 Directorate of Health Services, Government of Kerala, Thiruvananthapuram, 695 011 India; 7 Low Level Radiation Research Section (LLRRS), Homi Bhabha National Institute (HBNI), Radiation Biology & Health Sciences Division (RB&HSD), Bio-Science Group (BSG), Bhabha Atomic Research Centre (BARC), Mumbai, 400 085 India; 8 Homi Bhabha National Institute, Anushaktinagar, Mumbai, 400 094 India

**Keywords:** High level natural radiation area (HLNRA), Normal level natural radiation areas (NLNRA), Prevalence at birth, Hospital-based newborn monitoring, Congenital heart disease, Chronic low dose radiation

## Abstract

**Background:**

The human population residing in monazite bearing Kerala coast are exposed to chronic low dose and low dose rate external gamma radiation due to Th232 deposits in its beach sand. The radiation level in this area varies from < 1.0 to 45.0 mGy/year. This area serves as an ideal source for conducting large-scale epidemiological studies for assessing risk of low dose and low dose rate radiation exposure on human population. The areas with a dose level of ≤1.50 mGy/year are considered as normal level natural radiation areas (NLNRAs) and areas with > 1.50 mGy/year, as high level natural radiation areas (HLNRAs). HLNRAs were further stratified into three dose groups of 1.51-3.0 mGy/year, 3.01-6.00 mGy/year and > 6.0 mGy/year. The present study evaluates the effects of chronic low dose radiation (LDR) exposure on the birth prevalence of Congenital Heart Diseases (CHD) among the live newborns monitored in hospital based prospective study from NLNRAs and HLNRAs of Kerala coast, India.

**Methodology:**

Consecutive newborns were monitored from two hospital units located in the study area for congenital malformations. Referred CHD cases among the newborns screened were confirmed by conducting investigations such as pulse oximetry, chest X-ray, electrocardiogram and echocardiogram etc.

**Results:**

Among the newborns screened, 289 CHDs were identified with a frequency of 1.49‰ among 193,634 livebirths, which constituted 6.03% of overall malformations and 16.29% of major malformations. Multiple logistic regression analysis suggested that the risk of CHD among the newborns of mothers from HLNRAs with a dose group of 1.51-3.0 mGy/year was significantly lower as compared to NLNRA (OR = 0.72, 95% CI: 0.57-0.92), whereas it was similar in HLNRA dose groups of 3.01-6.00 mGy/year (OR = 0.55, 95% CI: 0.31-1.00) and ≥ 6.0 mGy/year (OR = 0.96, 95% CI: 0.50-1.85). The frequency of CHDs did not show any radiation dose related increasing trend. However, a significant (*P* = 0.005) reduction was observed in the birth prevalence of CHDs among the newborns from HLNRA (1.28‰) as compared to NLNRA (1.79‰).

**Conclusion:**

Chronic LDR exposure did not show any increased risk on the birth prevalence of CHDs from high-level natural radiation areas of Kerala coast, India. No linear increasing trend was observed with respect to different background dose groups. The frequency of CHD was observed to be 1.49 per 1000 livebirths, which was similar to the frequency of severe CHD rate reported elsewhere in India and was much less than the reported frequency of 9 per thousand.

## Introduction

Congenital malformations are structural, functional or cosmetic disabilities present at the time of birth. They develop prenatally and are identified at the time of birth or before or after birth. Congenital heart diseases (CHD) are one of the most common major birth defects, which can involve the walls, valves, arteries or veins of the heart. The birth prevalence of CHD varies widely worldwide and ranges from 0.6 to 75 per 1000 live births [1–4] with consistent increase after the introduction of echocardiography and reporting of even minor form of CHD. In India, large-scale studies on the prevalence of CHD at birth are limited and available reports of birth prevalence of CHD range from 3 to 13 per 1000 live births and sometimes found to be as high as 74, if minor form of CHDs are included [5]. The reported birth prevalence of severe CHD, requiring an intervention in the first year of life itself, is 1.5-1.7 per 1000 live births [5].

About 70-80% of CHD cases are multifactorial in origin, which include interaction of both genetic and environmental factors [1]. Several investigations involving advanced techniques like fluorescence-in-situ hybridization (FISH), Comparative genomic hybridization (CGH), exome sequencing etc., reveal that aneuploidy, gene mutations, copy number variations and single nucleotide polymorphisms are associated with CHD [1, 6]. There are several factors that are reported to be associated with CHD including use of anticonvulsant medicines, lithium, alcohol, tobacco etc. In addition, maternal illnesses such as uncontrolled insulin dependent diabetic mellitus, systemic lupus erythematosus, rubella infection etc., in the first trimester of pregnancy [7] also contribute substantially to the increase incidence of CHDs.

Ionizing radiation (IR) has clastogenic, teratogenic and carcinogenic potential for induction of somatic and germinal mutations. Both paternal and/or maternal exposure may induce germline mutations in the parental lineage. The induced mutations may have its impact during conception, pre-implantation, organogenesis and general intra-uterine development [8, 9], which may lead to congenital malformations including CHDs at birth. However, till date no significant increase in birth defects or hereditary anomalies are reported in human due to IR below 100 mSv [10].

The risk of parental exposure to IR below 100 mSv on birth prevalence of CHD in human population is not clearly established. Linear No Threshold (LNT) hypothesis, commonly used as basis for regulatory and safety purposes, considers any dose of IR is harmful whether it is acute or chronic, low or high dose. Human population exposed to IR provides direct evidence on hereditary effects or cancer incidence. There are large scale epidemiological studies such as Atomic Bomb (A-Bomb) survivors, Chernobyl and Fukushima Daiichi nuclear disaster affected population, population exposed to occupational and medical exposures and most importantly, those residing in HLNRAs of the world such as Yangjiang (china), Guarapari (Brazil), Ramsar (Iran) and Kerala (India). Even the largest epidemiological study involving children of survivors of the atomic bombings (A-Bomb) in Japan did not indicate any significant impact of acute radiation exposures in human population [11]. No statistically significant increase in major birth defects or other untoward pregnancy outcomes was observed among children of A-Bomb survivors. Similarly, studies conducted in Chernobyl nuclear disaster areas did not show any increase in the birth prevalence of congenital malformations [12]. Congenital Heart Disease after the Fukushima nuclear accident also did not show any increase in CHDs [13].

Although the transgenerational and intra-uterine effect of LDR on the development of heart disease is poorly understood, somatic effect of IR was recognized in late 1960s [14] when thoracic radiation exposure was identified as a risk factor. Several epidemiological studies including studies on Japanese A-Bomb survivors [15, 16] and studies on occupational exposures on human population have consistently suggested positive relationship between IR and cardiovascular diseases, for doses > 0.5 Gy as reported by Baselet et al. [17] Epidemiological studies require very large sample size, up to several millions to detect the risk of CHDs at low doses, especially for dose levels of < 0.5 Gy with sufficient statistical power [17]. Hence, studies on birth prevalence of CHDs among a large population exposed to LDR exposure in HLNRA of Kerala coast, provide a practical approach to shed light on this area of research, especially in the context of recent interest and debate on the effect of LDR exposure on circulatory diseases as a whole [18].

The study area is a narrow coastal belt in the state of Kerala (55 km long and 0.5 km wide) in southwest India extending from Neendakara (Kollam district) in the south to Purakkad (Alappuzha district) in the north. It has a patchy distribution of monazite in its beach sand that contains Th-232, and its decay products. Due to the uneven distribution of monazite in the beach sand, the level of radiation exposure varies extensively in this area, which ranges from < 1.0 to 45 mGy/year [19, 20]. The level of radiation exposure is elevated from place to place and in some places, it is above 70 mGy/year. The human population residing in this area is more than 1000 years old. This population is exposed to chronic LDR exposure and provides useful information on epidemiological and biological endpoints due to elevated level of background radiation at all stages of development. Screening of newborns of the HLNRA and adjacent NLNRA of Kerala is being carried out to assess the risk of congenital malformations (hereditary risk) among the offspring of parents exposed to LDR, projected to be about 0.3 to 0.5% per Gy to the first generation [10]. So far, epidemiological data on congenital malformations and cytogenetic investigations on newborns did not reveal any increased frequency in any of the parameters in HLNRA as compared to NLNRA [21–26]. The present investigation is focused on the effect of chronic LDR effects on birth prevalence of CHDs from newborn survey conducted in HLNRA of Kerala coast.

The objective of the present study is to delineate the risk and/or effect of chronic LDR exposure on CHDs among the newborns from HLNRAs and the adjoining NLNRAs of Kerala coast.

## Materials and methods

Screening of the newborns in hospital units located in NLNRA and HLNRA is described in detail elsewhere [21, 24]. Briefly, the study was carried out in selected government hospitals catering to the medical needs of the population residing in the HLNRA and adjacent NLNRA under Memorandum of Understanding (MoU) between Bhabha Atomic Research Centre (BARC) and Department of Health and Family Welfare, Directorate of Health Services, Government of Kerala. All the women admitted for delivery with a gestational age of more than 28 weeks were included in the study and the newborn infants, alive or dead, were the primary study subjects. Data related to the socio-economic profile, pregnancy history, life style, occupation, place of stay of the parents, etc. were collected in pre-coded proforma along with the details of the current pregnancy outcome. Congenital anomaly was identified primarily by clinical examination of the newborns by paediatricians of the collaborative hospitals and was further verified by trained medical doctors of Low-Level Radiation Research Laboratory (LLRRL), Kollam, who made regular visits to the participating hospitals. Newborns showing signs and symptoms of heart defects on clinical evaluation were examined by pulse oximetry, electrocardiogram, chest x-ray and echocardiogram for diagnosing CHD.

### Dosimetry

The study area was divided into small grids of 100 m^2^ and radiation dose level of each grid was estimated by taking the mean of several survey meter (halogen-quenched Geiger Muller tube-based survey meter consisting of the tube and microprocessor based digital display) readings taken across the length and breadth of the grid at 1 m height from the ground and the dose was assigned to all the newborns with parental residence in the grid. Areas with a radiation exposure level above 1.5 mGy/year were considered as HLNRA and those below 1.50 mGy/year, as NLNRA [21].

### Statistical methods

Chi-square test, with Yate’s continuity correction where indicated, was used to compare the frequencies of CHD across different sub-groups. Multiple logistic regression using STATISTICA software [27] was employed to estimate the risk of CHD in terms of Odds Ratio (OR). To estimate the contribution of paternal age to the risk of CHD, taking into account the maternal age effect and the high correlation between maternal and paternal age, simple logistic regression was used with parental age difference (age of father minus age of mother, ignoring negative values) as independent variable, separately in the maternal age groups of 15-19, 20-21, 22-23, 24-25, 26-27, 28-29 and ≥ 30 years. Parental age difference was grouped as 0-4, 5-9 and ≥ 10 years, with 0-4 years as the reference category. The statistical significance of OR was judged from its 95% CI, a 95% CI that do not include the null value of 1, suggests statistical significance of the OR at 5% level. No correction for multiple testing was carried out.

## Results

During the period from August 1995 till December 2020, a total of 194,167 newborns were monitored from 192,822 deliveries, which include 1318 twins, 12 triplets and one quadruplet in hospital units of NLNRA and HLNRA of Kerala coast. Age at birth of the mothers ranged from 15 to 45 years with a mean age of 24.7 ± 3.8 years and about 83% were in the age group of 20-29 years. Majority (~ 92%) of the births was either from first (46.2%) or second (45.6%) gravida/delivery. The overall sex-ratio of the newborns monitored was 1042 males to 1000 females. Among these, congenital malformations were detected in 4668 (24.04‰) newborns of which 1774 (9.14‰) had major congenital malformations. About 59% (114,764) newborns were from HLNRA and 41% (79,403), from NLNRA. Major malformations (0.92% v/s 0.91%), stillbirths including born alive but died immediately after birth (0.38% v/s 0.34%), down syndrome (0.065% v/s 0.065%) and live singleton newborns with low birthweight of < 2500 g (8.13% v/s 8.15%) were observed to be similar in HLNRA and NLNRA, respectively. The prevalence of paternal smoking habit was 35% and it was not associated with the risk of any of the end-points considered. Maternal smoking was rare (< 1%) in the study population.

A total of 533 infants were born dead, either as still birth or intrauterine death and were excluded from the analysis of congenital heart disease (CHD) among the live births. Among 193,634 live newborns, a total of 289 newborns were identified as CHDs (138 males; 151 females) with a frequency 1.49‰ of which 23 newborns with CHD (15 males; 8 females) died within few days without any physical stress. Though male newborns with CHD died immediately after birth, which is twice as often as their female counterpart, the difference was not statistically significant (RR = 2.05, 95% CI: 0.90-4.69). CHD constituted 6.03% of overall malformations and 16.29% of major congenital malformations with serious structural, functional, or developmental disability. About 86% of the newborns had CHD alone and the remaining 14% had other major malformations involving central nervous, musculoskeletal, genitourinary, gastrointestinal, respiratory systems or syndromes.

Maternal age at birth, gravida status, gender of the newborns and radiation dose levels at the area of parental residence were found to be associated with overall as well as major congenital malformations. However, the variation observed in the frequency of malformations across different religious groups was well within the limits of random fluctuation (Table 1). While there was statistically significant difference in the frequency of overall congenital malformation among different ethnic groups within Hindu religious group, the frequency of major congenital anomalies was statistically similar among them (Table 1).

**Table 1:** Overall and major malformations and heart diseases among newborns according to maternal and newborn characteristics

	N	No. babies born dead	No. of newborns with			OR^e^ CHD (95% CI)
			CA^a^ (‰)	MCA^b^ (‰)	CHD^c^ (‰^d^)	
**Maternal age at birth in years**						
15-19	11,886	33	255 (21.45)	93 (7.82)	15 (1.27)	0.87 (0.49-1.54)
20-21	29,068	74	693 (23.84)	246 (8.46)	25 (0.86)	0.59 (0.37-0.94)^h^
22-23	41,424	95	957 (23.1)	374 (9.03)	61 (1.48)	1.00
24-25	40,533	115	988 (24.38)	349 (8.61)	57 (1.41)	0.95 (0.66-1.37)
26-27	29,905	84	714 (23.88)	279 (9.33)	54 (1.81)	1.22 (0.84-1.77)
28-29	19,938	50	485 (24.33)	189 (9.48)	36 (1.81)	1.21 (0.79-1.85)
≥ 30	21,413	82	576 (26.9)	244 (11.39)	41 (1.92)	1.26 (0.83-1.91)
			*P*^f^ = 0.047	*P* = 0.008	*P* = 0.026	
**Gravida Status**						
1	89,787	234	2236 (24.9)	784 (8.73)	119 (1.33)	1.00
2	88,482	205	2060 (23.28)	818 (9.24)	140 (1.59)	1.02 (0.78-1.33)
3	13,826	71	313 (22.64)	139 (10.05)	23 (1.67)	1.02 (0.63-1.64)
≥ 4	2072	23	59 (28.47)	33 (15.93)	7 (3.42)	2.02 (0.92-4.44)
			*P* = 0.048	*P* = 0.004	*P* = 0.057	
**Gender of the newborn**^**g**^						
Male	99,085	287	2882 (29.09)	1048 (10.58)	138 (1.40)	0.88 (0.70-1.10)
Female	95,060	243	1764 (18.56)	704 (7.41)	151 (1.59)	1.00
			*P* < 0.001	*P* < 0.001	*P* = 0.291	
**Consanguinity**						
No	190,879	516	4579 (23.99)	1723 (9.03)	283 (1.49)	1.00
Yes	3288	17	89 (27.07)	51 (15.51)	6 (1.83)	1.31 (0.58-2.96)
			*P* = 0.253	*P* < 0.001	*P* = 0.778	
**Religion and Ethnicity**						
Hindu	132,138	367	3190 (24.14)	1179 (8.92)	190 (1.44)	–
Nair	26,791	66	588 (21.95)	229 (8.55)	41 (1.53)	1.00
Ezhava	48,688	121	1251 (25.69)	442 (9.08)	70 (1.44)	0.93 (0.63-1.37)
Viswakarma	13,656	27	348 (25.48)	118 (8.64)	21 (1.54)	0.96 (0.57-1.63)
Others	43,003	153	1003 (23.32)	390 (9.07)	58 (1.35)	0.86 (0.58-1.29)
Christian	21,724	61	532 (24.49)	201 (9.25)	39 (1.8)	1.14 (0.74-1.78)
Muslim	40,305	105	946 (23.47)	394 (9.78)	60 (1.49)	1.01 (0.67-1.51)
			*P* = 0.670	*P* = 0.284	*P* = 0.449	
**Radiation dose in mGy/year**						
≤ 1.50	79,403	203	2059 (25.93)	721 (9.08)	142 (1.79)	1.00
1.51-3.00	96,393	290	2211 (22.94)	901 (9.35)	125 (1.3)	0.72 (0.57-0.92)^h^
3.01-6.00	12,256	25	260 (21.21)	89 (7.26)	12 (0.98)	0.55 (0.31-1.0)
≥ 6.01	6115	15	138 (22.57)	63 (10.3)	10 (1.64)	0.96 (0.5-1.85)
			*P* < 0.001	*P* = 0.103	*P* = 0.024	
Total	194,167	533	4668 (24.04)	1774 (9.14)	289 (1.49)	

N=No. of total births

^a^CA – congenital anomaly

^b^MCA – major congenital anomaly

^c^CHD – congenital heart disease

^d^CHD frequency calculated per 1000 Live births

^e^Odds Ratio, with 95% Confidence Interval obtained by employing multiple logistic regression with all the characteristics in the model and category with OR as 1 as reference

^f^P-values of chi-square test comparing the frequencies across different sub-groups after applying Yate’s correction, where necessary

^g^There were 22 cases with intersex, of which 3 were born dead

^h^Statistically significant

Detailed analysis of the frequency of congenital heart disease (CHD) among live births was carried out among the newborns monitored. CHD was least frequent in the maternal age group of 20-21 years (0.86‰) and most frequent (1.92‰) in the age group of ≥30 years (Table 1). Chi-square test suggested that the associations of maternal age at birth with CHD was statistically significant (*P* = 0.026) with increase in CHD after the maternal age at birth of 26 years and a statistically significant maternal age at birth related linear trend (Trend χ^2^ = 10.7, *P* < 0.01). Newborns of mothers with gravida status of 4 or more had the highest frequency of CHD (3.42‰) as compared to 1.33‰, 1.59‰ and 1.67‰ among mothers with gravida status of 1, 2 and 3, respectively (*P* = 0.057). Female newborns had relatively higher frequency of CHD (1.59‰) as compared to male newborns (1.40‰), but the difference was not statistically significant (*P* = 0.291). Newborns from consanguineous (1.83‰) and non-consanguineous marriage groups (1.49‰) had statistically similar frequency of CHD (*P* = 0.778). The variation in the CHD frequency across the three religious groups i.e., Hindu, Christian and Muslim as well as that among different ethnic groups within Hindu religious group was within the limits of chance fluctuations/ occurrences.

The data was stratified based on background radiation level at the area of parental residence and was grouped as ≤1.50, 1.51-3.0, 3.01-6.0 and > 6.0 mGy/year. The CHD rates among the newborns of mothers belonging to these areas were 1.79‰, 1.30‰, 0.98‰ and 1.64‰ respectively (Table 1). Chi-square test suggested that the difference in the incidence of CHDs in the four radiation dose groups to be statistically significant (*P* = 0.024). The overall frequency of CHD in HLNRA was 1.28‰, which was significantly (*P* = 0.005) different from the frequency 1.79‰ observed from NLNRA.

The odds ratio (OR) was estimated using multiple logistic regression analysis to assess the individual contributions of maternal age at birth, gravida status, gender of the newborn, consanguinity, religion and ethnicity together with background radiation dose to the risk of CHD and is depicted in Table 1 with OR of 1 indicating reference category. The analysis suggested that maternal age at birth and radiation levels at the area of parental residence is associated with CHD. The risk of CHD was less in the age group of 20-21 years as compared to the reference group of 22-23 years (OR = 0.59, 95% CI: 0.37-0.94). The risk of CHD in all the other age groups was statistically similar to that in the age group of 22-23 years. The risk of CHD among newborns from areas with background dose levels of 1.51-3.0 mGy/year was significantly lower as compared to NLNRA (OR = 0.72, 95% CI: 0.57-0.92). The risk was statistically similar in the dose group of ≤1.50 mGy/year and in the HLNRA dose groups of 3.01-6.0 mGy/year (OR = 0.55, 95% CI: 0.31-1.00) and ≥ 6.0 mGy/year (OR = 0.96, 95% CI: 0.50-1.85).

**Fig. 1:**
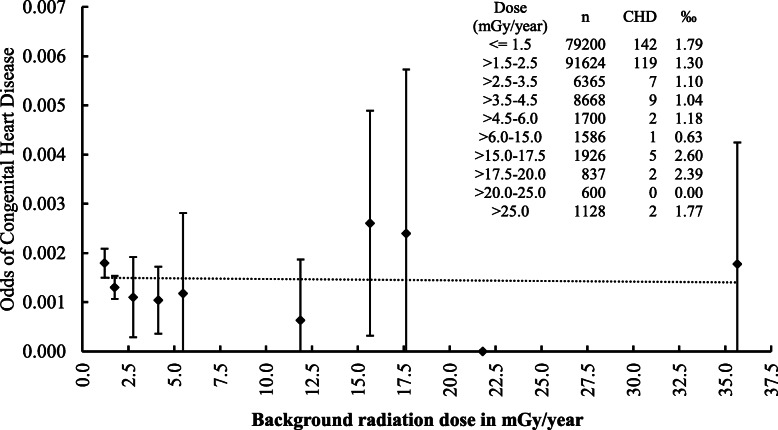
Odds of congenital heart disease (CHD) among live newborns with +/− 95% CI by geometric mean dose in dose categories fitted with logistic regression estimate of the odds (dotted line). CHD odds ratio per mGy = 0.998, *P* = 0.912

The background dose at the parental residence was further subdivided to assess the dose response of CHD with respect to radiation dose. The odds of CHD in each dose group together with its 95% CI is depicted in Fig. 1. The line of best linear fit of the odds, estimated using logistic regression by using the geometric mean of the doses in each dose group, does not seem to suggest any trend in the risk of CHD with increasing background dose. An apparent statistically non-significant reduction was observed in the first few dose groups of 1.51-2.50, 2.51-3.50, 3.51-4.50, 4.51-6.0 and 6.01-15.0 mGy/year relative to that in ≤1.50 mGy/year, the NLNRA.

### Effect of paternal age on CHD

**Fig. 2:**
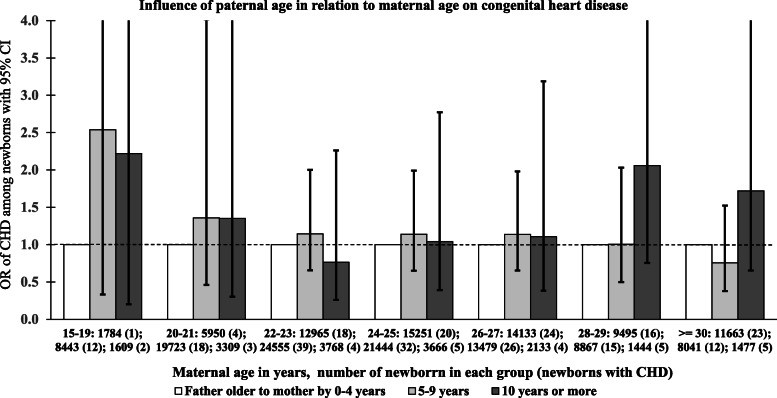
Influence of paternal age at birth on the risk of congenital heart disease. The risk was estimated among newborns with paternal age more than the maternal age by 5-9 years & ≥10 years relative to those who were within 4 years, separately within each maternal age group. Horizontal axis gives the maternal age group and number of newborns in each group, grouped by the difference between parental age, together with number of newborns with CHD in parenthesis

Of the 193,634 live newborns, paternal age was not available for 55 cases and it was less than maternal age in 380 (0.2%) cases. Hence, analysis was done on 193,199 newborns to find out the influence of paternal age at birth. As maternal age and paternal age were highly correlated, the effect of paternal age at birth was analysed by grouping the newborns whose paternal age is more than the maternal age by 4 years or less (36.9%), 5-9 years (54.1%) and ≥ 10 years (9%). The analysis was carried out separately in the maternal age groups of 15-19, 20-21, 22-23, 24-25, 26-27, 28-29 and ≥ 30 years. The risk of CHD among newborns whose paternal age is more than the maternal age by 5-9 years and ≥ 10 years relative to those who were within 4 years was assessed by using odds ratio. The results as depicted in Fig. 2, do not seem to suggest any statistically significant influence of paternal age at birth, though mothers aged 28 or more appear to have a higher risk of having a newborn with CHD, if the paternal age is greater by 10 years or more. The influence of paternal age at birth seems to be minimal for maternal age at birth of 27 years or less.

## Discussion

The present study is a part of the newborn monitoring programme in the HLNRAs of Kerala coast, India to assess the influence of natural chronic low dose and low dose rate IR exposure directly on birth defects on offspring at birth. The study evaluated the symptomatic cases of CHD among consecutive new-borns at birth, detected by clinical examination of the baby and/or by further clinical investigations, where necessary. The study population has a high literacy rate and practices small family norms, where more than 90% of the mothers had a gravida status of 1 or 2. More than 80% of mothers had an age at delivery or child birth between 20 and 29 years with almost fully institutionalized delivery. Other reproductive parameters monitored during delivery was comparable to developed countries [24, 26]. The birth prevalence of CHD observed in the study was 1.49 per 1000 births, is rather similar to the severe CHD frequency rate reported elsewhere in India and is less than 9 per 1000 live births reported to be a representative figure [5]. As the study monitored newborns at birth and only detectable CHDs were identified, there is a chance of missing minor forms of CHDs and CHDs identified during infancy and later. This would perhaps explain the lower frequency rate observed in the study population as compared to that observed across India ranging from 3.9-9.0/1000 births [5, 28]. The number of newborns with CHDs, who died within first few days of hospital stay of the mother (3 days after normal vaginal delivery and 7 days after caesarean section) was 8% (23 out of 289 newborns diagnosed with CHD) in our study, which is much less than the reported figure of 29% [29]. The aetiology of CHD is not fully understood, though maternal, genetic and environmental factors have been implicated [1, 6, 7].

A study based on acute radiation exposure from the Chernobyl accident has not found any correlation between radioactive contamination and CHDs [30]. In our study, chronic LDR does not show any positive correlation on CHDs. On the contrary, the prevalence of CHDs among newborns of mothers hailing from areas within the dose group of 1.51-3.0, 3.01-6.0 and > 6.0 mGy/year were lower as compared to the dose group of ≤1.50 mGy/year (NLNRA) and with statistically significant reduction in the dose group of 1.51-3.00 mGy/year (OR = 0.72; 95% CI: 0.57-0.92) without any dose related trend in the incidence of CHDs (Fig. 1).

The heart is considered as a radio-resistant organ, which is not influenced by doses up to 30 Gy [31]. High doses of IR from medical exposures may increase the severity of heart diseases [17]. An elevated risk for heart diseases was reported from Life span study (LSS) from Japan (ERR = 0.14 per Gy, 95% CI: 0.06-0.23), but the dose-response relationship below 0.5 Gy was not statistically significant [16]. Chernobyl liquidator cohort yielded an ERR/Gy of 0.41 for Ischemic Heart Disease (IHD) with 95% CI: 0.05-0.78, whereas Mayak cohort yielded an ERR/Gy of 0.11 with 95% CI: 0.049-0.168 [32] and reported a statistically significant female-workers-influenced decrease in incidence of IHDs among the workers exposed to external doses of 0.2 to 0.5 Gy as compared to workers exposed to external doses below 0.2 Gy [33]. The relationship between radiation exposure and heart disease may be obscured by the extrapolation of the findings from cancer patients to general population in cancer survivor studies, by ‘healthy survivor’ effect in LSS cohort and ‘healthy worker’ effect in occupational exposure studies.

The overall statistically significant reduction in the risk of CHDs in HLNRA as compared to NLNRA and the apparent reduction, though not statistically significant, at the dose levels of 1.51-2.50, 2.51-3.50, 3.51-4.50, 4.50-6.0 and 6.01-15.0 mGy/year (as shown in Fig. 1) is noteworthy. The observation is relevant in the context of recent discussions on the validity and veracity of the LNT hypothesis and the suggestion of a possible beneficial effect, especially in the low dose region [34–36].

The contribution of paternal age towards congenital malformation and CHD in particular is equivocal or weak [37] while that of maternal age is well known fact as it is established that advanced maternal age has impact on congenital malformations in general and CHD in particular. The reason could be due to the difference in the gametogenesis of males and females. Male gametic stem cells undergo meiosis continuously after puberty while there is a long gap between first meiotic division during female fetal development and second meiosis at fertilization [9]. Unravelling the effect of paternal age from maternal age is tricky as the maternal and paternal age are highly correlated in the study (*r* = 0.762) population and paternal age is usually greater than maternal age (99.8%). It may not be possible to vary one ‘keeping the other constant’ so as to apply regression techniques to estimate the individual contribution of maternal and paternal age on CHD. Hence, it was attempted to describe the effect of paternal age by comparing the risk of CHD among newborns whose paternal age was more than the maternal age by 5-9 years and ≥ 10 years relative to those who were within 4 years (Fig. 2), separately within each maternal age group [38].

The present study population, which is exposed to high level natural background radiation exposure to human population at all stages of human development is of high relevance to assess the risk of chronic LDR exposure below 100 mSv (mGy). Moreover, the availability of large sample size of newborns monitored in NLNRA and HLNRA in Kerala coast for the last three decades for delineating the effect of chronic LDR exposure on CHD is noteworthy. To our knowledge this is one of the largest studies on newborns in the World, where the risk of LDR exposure in human population and the offspring is assessed. No such data is available on birth prevalence of CHDs in a larger cohort of newborn population to delineate the effect of LDR.

### Strength and limitations of the study

This study is based on monitoring 194,167 consecutive newborns at birth and the large sample size is the major strength of this study. The chance of under ascertainment of severe CHDs detectable at birth was remote in the study since all newborns were examined by the paediatricians of the collaborating hospitals and their observations were verified by medical doctors of LLRRL, BARC trained in detecting congenital anomalies. All the relevant concomitant information was recorded in pre-coded proforma during the stay of the woman in the hospital by staff nurses and medical doctors of the hospital. The data were scrutinized for completeness and consistency by medical and paramedical staff of LLRRL, who made regular visits to the participating hospitals. The uniqueness of the study population such as lower maternal age at birth, acceptance of small family norms, universal antenatal folic acid supplementation, higher literacy rate mediated health awareness and practices may have influence in reducing the prevalence of major cardiovascular anomalies. As only suspected cases of CHDs were referred for further investigation, chance of missing minor CHDs may not be ruled out. Radiation exposure profile in the natural high-level radiation scenario is quite complex due to local movement of the population coupled with the general Indian pattern of shift of residence of female to her husband’s place after marriage. One of the limitations of the study is that the average dose at the area of residence of the parents was used in the study as a surrogate for the actual dose. Since the radiation level in the region under study is purely due to primordial radioactivity, drastic change in radiation levels over the years was unlikely. Smaller sample size from the areas with higher radiation levels was another limitation of this study, which is the characteristics of this population.

## Conclusion

In conclusion, the study revealed that natural chronic low dose and low dose radiation exposure did not increase the incidence of CHD in HLNRA of Kerala coast as compared to the adjacent NLNRA. On the contrary, a reduction in the birth prevalence of CHDs were observed among the newborns of mothers hailing from areas with HLNRA (1.28‰) as compared to those from normal level natural radiation areas (1.79‰), *P* = 0.005. There was no increasing trend with respect to chronic low dose and low dose background radiation exposure to this population.

## Data Availability

The dataset used and analyzed during the current study are available from the corresponding author, on reasonable request.

## Abbreviations

LDR: Low dose radiation
IR: Ionizing radiation
mGy: Milli Gray
CHD: Congenital heart disease
HLNRA: High level natural radiation area
NLNRA: Normal level natural radiation area
FISH: Fluorescence-in-situ hybridization
CGH: Comparative genomic hybridization
IHD: Ischemic Heart Disease
A-Bomb: Atomic Bombings
LNT: Linear No Threshold
LSS: Life span study
ERR: Excess relative risk
OR: Odds ratio
CI: Confidence Interval

## References

[1] Richards AA, Garg V (2010). Genetics of congenital heart disease. Curr Cardiol Rev.

[2] der Linde V, Konings EEM, Slager MA, Witsenburg M, Helbing WA, Takkenberg JJM, Roos-Hessenlink JW (2011). Birth prevalence of congenital heart disease worldwide: a systematic review and meta-analysis. J Am Coll Cardiol.

[3] Hoffman JIE, Kaplan S (2002). The incidence of congenital heart disease. J Am Coll Cardiol.

[4] Hoffman JIE (2013). The global burden of congenital heart disease. Cardiovasc J Afr.

[5] Saxena A (2018). Congenital heart disease in India; a status report. Indian Pediatr.

[6] Ekure EN, Adeyemo A, Liu H, Sokunbi O, Kalu N, Martinez AF, Owosela B, Tekendo-Ngongang C, Addissie YA, Olusegun-Joseph A, Ikebudu D, Berger SI, Muenke M, Han Z, Kruszka P (2021). Exome sequencing and congenital heart disease in sub-Saharan Africa. Circ Genom Precis Med.

[7] Jenkins KJ, Correa A, Feinstein JA, Botto L, Britt AE, Daniels SR, Elixson M, Warnes CA, Webb CL (2007). Noninherited risk factors and congenital cardiovascular defects: current knowledge: a scientific statement from the American Heart Association Council on Cardiovascular Disease in the Young: endorsed by the American Academy of Pediatrics. Circulation..

[8] Coggle JE (1983). Biological effects of radiation.

[9] Vogel F, Motulsky AG (1997). Human genetics - problems and approaches.

[10] United Nations Scientific Committee on the Effects of Atomic Radiation (2001). Report to the general assembly with scientific annex: hereditary effects of radiation.

[11] Yamada M, Furukawa K, Tatsukawa Y, Marumo K, Funamoto S, Sakata R, et al. Congenital malformations and perinatal deaths among the children of atomic bomb survivors: a reappraisal. Am J Epidemiol. 2021:kwab099. 10.1093/aje/kwab099. 10.1093/aje/kwab099 PMC8561127 33847738

[12] Little J (1993). The Chernobyl accident, congenital anomalies and other reproductive outcomes. Paediatr Perinat Epidemiol.

[13] Hirata Y, Shimizu H, Kumamaru H, Takamoto S, Motomura N, Miyata H, Okita Y (2020). Congenital heart disease after the Fukushima nuclear accident: the Japan cardiovascular surgery database study. J Am Heart Assoc.

[14] Stewart JR, Fajardo LF (1971). Radiation-induced heart disease. Clinical and experimental aspects. Radiol Clin N Am.

[15] Schultz-Hector S, Trott KR (2007). Radiation-induced cardiovascular diseases: is the epidemiologic evidence compatible with the radiobiologic data?. Int J Radiat Oncol Biol Phys.

[16] Shimizu Y, Kodama K, Nishi N, Kasagi F, Suyama A, Soda M, Grant EJ, Sugiyama H, Sakata R, Moriwaki H, Hayashi M, Konda M, Shore RE (2010). Radiation exposure and circulatory disease risk: Hiroshima and Nagasaki atomic bomb survivor data, 1950-2003. BMJ..

[17] Baselet B, Rombouts C, Benotmane AM, Baatout S, Aerts AN (2016). Cardiovascular diseases related to ionizing radiation: the risk of low-dose exposure (review). Int J Mol Med.

[18] Little MP, Azizova TV, Bazyka D, Bouffler SD, Cardis E, Chekin S, Chumak VV, Cucinotta FA, de Vathaire F, Hall P, Harrison JD, Hildebrandt G, Ivanov V, Kashcheev VV, Klymenko SV, Kreuzer M, Laurent O, Ozasa K, Schneider T, Tapio S, Taylor AM, Tzoulaki I, Vandoolaeghe WL, Wakeford R, Zablotska LB, Zhang W, Lipshultz SE (2012). Systematic review and meta-analysis of circulatory disease from exposure to low-level ionizing radiation and estimates of potential population mortality risks. Environ Health Perspect.

[19] Bharatwal DS, Vaze GH (1959). Radiation dose measurements in the monazite areas of Kerala state in India. Proceedings of the 2ndUN international conference on peaceful uses of atomic energy.

[20] Sunta CM, Sohrabi M, Ahmed JU, Durani SA (1993). A review of the studies of high background areas of the S–W coast of India. Proceedings of the international conference on high levels of natural radiation.

[21] Jaikrishan G, Andrews VJ, Thampi MV, Koya PKM, Chauhan PS (1999). Genetic monitoring of the human population from high level natural radiation areas of Kerala on the southwest coast of India. I. Prevalence of congenital malformations in newborns. Radiat Res.

[22] Cheriyan VD, Kurien CJ, Das B, Ramachandran EN, Karuppasamy CV, Thampi MV, George KP, Kesavan PC, Koya PKM, Chauhan PS (1999). Genetic monitoring of the human population from high-level natural radiation areas of Kerala on the southwest coast of India. II. Incidence of numerical and structural chromosomal aberrations in the lymphocytes of newborns. Radiat Res.

[23] Das B, Karuppasamy CV (2009). Spontaneous frequency of micronuclei among the newborns from high level natural radiation areas of Kerala in the southwest coast of India. Int J Radiat Biol.

[24] Jaikrishan G, Sudheer KR, Andrews VJ, Koya PKM, Madhusoodhanan M, Jagadeesan CK, Seshadri M (2013). Study of stillbirth and major congenital anomaly among newborns in the high level natural radiation areas of Kerala, India. J Commun Genet.

[25] Ramachandran EN, Karuppasamy CV, Cheriyan VD, Soren DC, Birajalaxmi Das V, Anilkumar PKM, Koya MS (2013). Cytogenetic studies on newborns from high and normal level natural radiation areas of Kerala in southwest coast of India. Int J Radiat Biol.

[26] Koya PKM, Jaikrishan G, Sudheer KR, Andrews VJ, Madhusoodhanan M, Jagadeesan CK, Das B (2015). Sex ratio at birth: scenario from normal- and high-level natural radiation areas of Kerala coast in south-west India. Radiat Environ Biophys.

[27] StatSoft, Inc (2010). STATISTICA (data analysis software system), version 9.1.

[28] Saxena A, Mehta A, Sharma M, Salhan S, Kalivani M, Ramakrishnan S, Juneja R (2016). Birth prevalence of congenital heart disease: a cross-sectional observational study from North India. Ann Pediatr Card.

[29] Kudaiberdiev TZ, Akhmedova IA, Imanalieva GA, Tursunbekova GT, Tilemanbetova KT. Frequency of detection of congenital heart diseases in different regions of Kyrgyz Republic. Heart Vessels Transpl. 2017. 10.24969/hvt.2017.13.

[30] Frasch GA (1993). Is the incidence of congenital heart defects influenced by the radioactive contamination of the environment due to the accident in Chernobyl? — results from an epidemiological survey on congenital malformations in the south-German state of Bavaria. Environmetrics.

[31] Darby SC, Cutter DJ, Boerma M, Constine LS, Fajardo LF, Kodama K, Mabuchi K, Marks LB, Mettler FA, Pierce LJ, Trott KR, Yeh ETH, Shore RE (2010). Radiation-related heart disease: current knowledge and future prospects. Int J Radiat Oncol Biol Phys.

[32] Azizova TV, Muirhead CR, Druzhinina MB, Grigoryeva ES, Vlasenko EV, Sumina MV, O'Hagan JA, Zhang W, Haylock RG, Hunter N (2010). Cardiovascular diseases in the cohort of workers first employed at Mayak PA in 1948-1958. Radiat Res.

[33] Azizova TV, Muirhead CR, Moseeva MB, Grigoryeva ES, Vlasenko EV, Hunter N, Haylock RG, O'Hagan JA (2012). Ischemic heart disease in nuclear workers first employed at the Mayak PA in 1948-1972. Health Phys.

[34] Sutou S (2016). A message to Fukushima: nothing to fear but fear itself. Genes Environ.

[35] Sutou S (2018). Low-dose radiation from A-bombs elongated lifespan and reduced cancer mortality relative to un-irradiated individuals. Genes Environ.

[36] Sutou S (2020). Black rain in Hiroshima: a critique to the life span study of A-bomb survivors, basis of the linear no-threshold model. Genes Environ.

[37] Yang Q, Wen SW, Leader A, Chen XK, Lipson J, Wal M (2007). Paternal age and birth defects: how strong is the association?. Hum Reprod.

[38] Su XJ, Yuan W, Huang GY, Olsen J, Li J (2015). Paternal age and offspring congenital heart defects: a national cohort study. PLoS One.

